# Propofol Regulates ER Stress to Inhibit Tumour Growth and Sensitize Osteosarcoma to Doxorubicin

**DOI:** 10.1155/2023/3093945

**Published:** 2023-01-27

**Authors:** Hua Wei, Xinhui Du, Huaping Zhao, Peipei Sun, Jianjun Yang

**Affiliations:** ^1^Department of Anesthesiology, Pain and Perioperative Medicine, The First Affiliated Hospital of Zhengzhou University, 1 East Jianshe Road, Erqi District, Zhengzhou, Henan 450052, China; ^2^Bone and Soft Tissue Department, The Affiliated Cancer Hospital of Zhengzhou University & Henan Cancer Hospital, Zhengzhou 450008, China

## Abstract

Osteosarcoma is the most common malignant bone tumour affecting children and young adults. The antitumour role of propofol, a widely used intravenous sedative-hypnotic agent, has been recently reported in different cancer types. In this study, we aimed to assess the role of propofol on osteosarcoma and explore the possible mechanisms. Propofol of increasing concentrations (2.5, 5, 10, and 20 *μ*g/ml) was used to treat the MG63 and 143B cells for 72 hours, and the CCK8 assay was applied to evaluate the tumour cell proliferation. Tumour cell migration and invasion were assessed with the transwell assay. The tumour cells were also treated with doxorubicin single agent or in combination with propofol to explore their synergic role. Differential expressed genes after propofol treatment were obtained and functionally assessed with bioinformatic tools. Expression of ER stress markers CHOP, p-eIF2*α*, and XBP1s was evaluated to validate the activation of ER stress response with western blot and qRT-PCR. The statistical analyses were performed with R v4.2.1. Propofol treatment led to significant growth inhibition in MG63 and 143B cells in a dose-dependent manner (*p* < 0.05). Osteosarcoma migration (MG63 91.4 (82–102) vs. 56.8 (49–65), *p* < 0.05; 143B 96.6 (77–104) vs. 45.4 (28–54), *p* < 0.05) and invasion (MG63 68.6 (61–80) vs. 32 (25–39), *p* < 0.05; 143B 90.6 (72–100) vs. 39.2 (26–55), *p* < 0.05) were reduced after propofol treatment. Doxorubicin sensitivity was increased after propofol treatment compared with the control group (*p* < 0.05). Bioinformatic analysis showed significant functional enrichment in ER stress response after propofol treatment. Upregulation of CHOP, p-eIF2*α*, and XBP1s was detected in MG63 and 143B secondary to propofol treatment. In conclusion, we found that propofol treatment suppressed osteosarcoma proliferation and invasion and had a synergic role with doxorubicin by inducing ER stress. Our findings provided a novel option in osteosarcoma therapy.

## 1. Introduction

Osteosarcoma is a highly malignant bone tumour affecting the extremities of children and young adults. Limb salvage surgery and anthracycline (such as doxorubicin)-based chemotherapy are the first-line treatment for osteosarcoma [1]. Despite recent developments in surgery [2, 3] and systemic treatment methods [4, 5], local recurrence and drug resistance still pose as significant challenges and lead to decreased overall survival. Thus, novel methods to deal with osteosarcoma progression and drug resistance are pressingly needed.

Propofol is a widely used intravenous sedative-hypnotic agent, and its antitumour role has recently been recognized. The use of propofol as general anaesthesia induction in gastric cancer showed significantly better survival compared with using etomidate in specific TNM stages [6]. In vivo studies with a colorectal cancer xenograft model of mice also showed a significant decrease in tumour development with propofol in comparison with sevoflurane under nonsurgical conditions [7]. Besides tumour development, propofol also has been proposed to reverse drug resistance of multiple chemotherapy agents such as cisplatin [8–10], docetaxel [11], 5-fluorouracil [12], and others [13] in multiple cancer types.

Molecular mechanisms regarding the role of propofol on cancer have been proposed. For example, propofol has been reported to suppress lung cancer tumorigenesis by modulating the circ-ERBB2/miR-7-5p/FOXM1 axis [14]. Other studies showed that propofol suppressed colorectal cancer development by the circ-PABPN1/miR-638/SRSF1 axis [15] and mediated pancreatic cancer cell activity through the repression of ADAM8 via SP1 [16, 17]. The current understanding of possible molecular mechanisms has been extensively reviewed [18, 19].

Endoplasmic reticulum (ER) is involved in the biosynthesis of lipids and proteins, and many factors or drugs could lead to “ER stress,” a state induced by the accumulation of misfolded and/or unfolded proteins [20]. ER stress-induced osteosarcoma cell death was extensively reported [21–26].

In this study, we aimed to assess the role of propofol on osteosarcoma and its sensitivity to doxorubicin and explore the possible mechanism. We performed experiments on the MG63 and 143B cell lines and found that propofol inhibited cell proliferation, migration, invasion, and sensitized tumour cells to doxorubicin therapy. Our data also proved the presence of ER stress and activation of UPR pathways under propofol treatment. Based on our findings, we proposed that propofol could inhibit osteosarcoma malignancy and promote sensitivity to doxorubicin via inducing ER stress.

## 2. Materials and Methods

### 2.1. Cell Culture

Human osteosarcoma cell line 143B and MG63 were purchased from American Type Culture Collection (ATCC, Shanghai, China) and cultured in DMEM with 10% FBS (Gibco) at 5% CO_2_ and 37°C. For propofol treatment, MG63 and 143B cells were treated with 2.5, 5, 10, and 20 *μ*g/ml propofol (Sigma-Aldrich, St. Louis, MO, USA) for 72 hours and then collected for the tests.

### 2.2. Quantitative Real-Time PCR

Total RNA was extracted using TRIzol reagent ((Thermo Fisher Scientific), and their relative complementary deoxyribose nucleic acid (cDNA) was synthesized with Primescript RT Reagent (TaKaRa, Tokyo, Japan). qRT-PCR was performed with StepOne Plus Real-Time PCR system (Applied Biosystems, Foster City, CA, USA) with SYBR® Premix Ex Taq™ Reagent (TaKaRa, Tokyo, Japan). The following primers were used for qRT-PCR: XBP1, forward: 5'-GGAGTTAAGACAGCGCTTGG-3', reverse: 5'-GCACCTGCTGCGGACTC-3'; GAPDH, forward: 5'-ACCACAGTCCATGCCATC-3', reverse: 5'-TCCACCCTGTTGCTG-3'. Gene expression was analysed using the 2^−ΔΔCt^ method.

### 2.3. CCK8 Assay

Cells in their logarithmic growth phase were seeded into a 96-well plate at a density of 1 × 10^4^ cells/well and incubated with the treatment. After 72 h, 10 *μ*l of CCK8 solution (Shanghai, China) was added to each well and incubated for another 4 hours at 37°C. The absorbance of each well was measured at 450 nm with a microplate reader. Each experiment was conducted with 5 biological repeats.

### 2.4. Transwell Assay

Briefly, cells were suspended in the serum-free medium and seeded in the chambers of 24-well plates with or without Matrigel precoating. At 48 h culture, the chambers were taken out and penetrating cells were fixed with 5% paraformaldehyde for 20 min and dyed with 0.1% crystal violet for 20 min. Penetrating cells in 5 randomly selected fields of each sample were captured for counting using a light microscope (magnification 20x).

### 2.5. Western Blot

Cells were lysed by RIPA Lysis buffer (Thermo Fisher Scientific), and total proteins were collected. After transferring to a polyvinylidene fluoride (PVDF) membrane (Millipore, Billerica, MA, USA), the membrane was blocked in 5% skim milk for 1 h and incubated with primary antibodies at 4°C overnight and secondary antibodies for 1 h. The primary antibodies used were rabbit antihuman CHOP (1 : 1000, sc793, Santa Cruz), rabbit antihuman eIF2*α* (1 : 1000, #9722, Cell Signaling Technology), rabbit antihuman p-eIF2*α* (1 : 1000, #9721, Cell Signaling Technology), and rabbit antihuman GAPDH (1 : 2000, #5174, Cell Signaling Technology). Bands were exposed to electrochemiluminescence (ECL).

### 2.6. Bioinformatic and Statistical Analysis

All statistical analyses were performed in R v4.2.1 (https://www.R-project.org/). Data were presented as mean ± SD. The *t*-test was used for analysing measurement data attributed to the normal distribution and homogeneity of variance. *p* < 0.05 indicated the significant difference.

## 3. Results

### 3.1. Propofol Treatment Led to Growth Inhibition in Osteosarcoma

**Figure 1:**
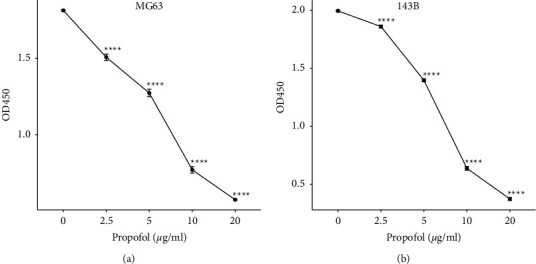
Propofol treatment led to growth inhibition in osteosarcoma. Increasing concentrations of propofol were used to treat the MG63 and 143B cells for 72 hours. Growth inhibition was shown in a dose-dependent manner in both (a) MG63 and (b) 143B cells. Each experiment was conducted with 5 biological repeats. ^*∗∗∗∗*^*p* < 0.001.

To assess whether propofol could affect osteosarcoma cell growth, the CCK8 assay was performed after propofol treatment. Increasing concentrations of propofol (2.5, 5, 10, and 20 *μ*g/ml) together with DMSO control were used to treat osteosarcoma cell lines MG63 and 143B for 72 hours. The remaining viable cells were assessed with the CCK8 assay. The reading of OD450 showed a gradual decrease with increasing concentrations of propofol in both cell lines (Figures 1(a) and 1(b)). The first dose (2.5 *μ*g/ml) already showed significant decrease compared to the control group in both cell lines (MG63 1.506 (1.440–1.556) vs. 1.812 (1.797–1.824), *p* < 0.05; 143B 1.859 (1.809–1.882) vs. 1.994 (1.957–2.016), *p* < 0.05). Those data showed that growth inhibition was observed in both cell lines in a dose-dependent manner. Since propofol (2.5 *μ*g/ml) showed a significant reduction in cell proliferation compared with the control group, it was used as the working concentration for the following experiments.

### 3.2. Propofol Treatment Inhibited Osteosarcoma Cell Migration and Invasion

**Figure 2:**
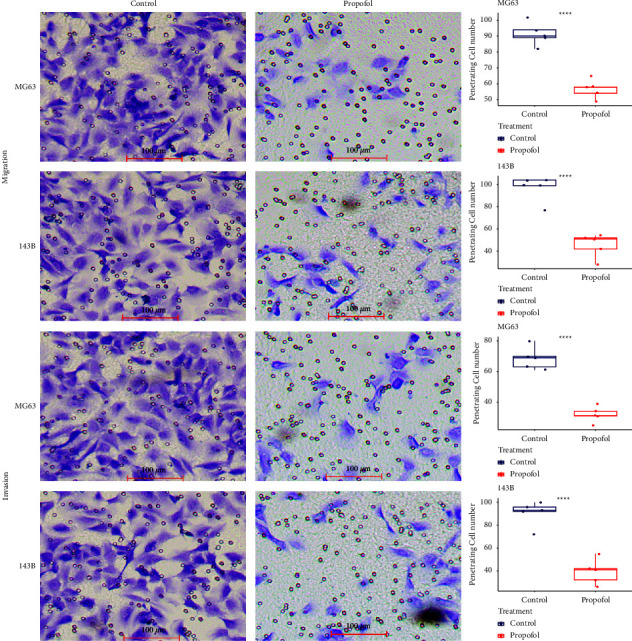
Propofol treatment led to decreased cell migration and invasion in MG63 and 143B cells. Each experiment was conducted with 5 biological repeats.

To investigate the role of propofol on cell migration and invasion, we performed the transwell assay on MG63 and 143B cell lines with propofol (2.5 *μ*g/ml) treatment (Figure 2). The number of both cell lines penetrating the chambers without Matrigel was significantly reduced after propofol treatment (MG63 91.4 (82–102) vs. 56.8 (49–65), *p* < 0.05; 143B 96.6 (77–104) vs. 45.4 (28–54), *p* < 0.05). The number of both cell lines penetrating the chambers with Matrigel was significantly reduced after propofol treatment (MG63 68.6 (61–80) vs. 32 (25–39), *p* < 0.05; 143B 90.6 (72–100) vs. 39.2 (26–55), *p* < 0.05). These data showed that propofol treatment inhibited osteosarcoma cell migration and invasion.

### 3.3. Propofol Sensitized Doxorubicin-Induced Growth Inhibition in Osteosarcoma

**Figure 3:**
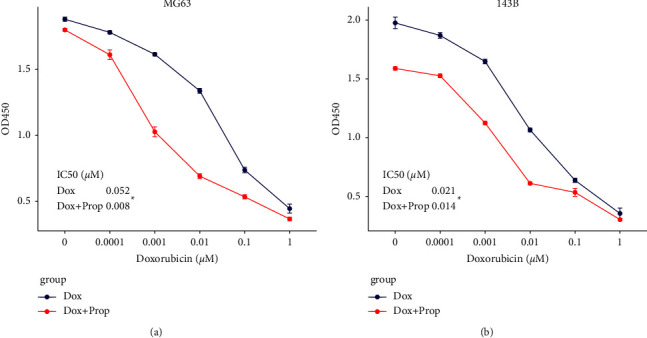
Propofol sensitized osteosarcoma to doxorubicin-induced growth inhibition. Doxorubicin single agent (increasing concentrations) or in combination with propofol (2.5 *μ*M) was used to treat the MG63 and 143B cells. The dose-response curves were plotted based on the remaining viable cell numbers and the IC50s were calculated in both conditions in the two cell lines. The IC50s were significantly lower in the combined therapy group than those in the doxorubicin single agent treatment group. Each experiment was conducted with 5 biological repeats. ^*∗*^*p* < 0.05.

Next, we aimed to explore whether propofol could affect the antitumour efficiency of doxorubicin in osteosarcoma. An increasing dose of doxorubicin were used to treat osteosarcoma cell lines MG63 and 143B with or without propofol (2.5 *μ*g/ml). The growth curves were plotted based on the OD450 readings, and the IC50s were calculated (Figure 3). The IC50 (*μ*M) of doxorubicin in combination with the propofol group was significantly lower than that of the doxorubicin single agent treatment group (0.008 vs 0.052, *p* < 0.05) in the MG63. Similar changes were also observed in 143B cells. The IC50 (*μ*M) was reduced from 0.021 in the doxorubicin single agent treatment group to 0.014 in the doxorubicin + propofol treatment group (*p* < 0.05). Based on these findings, we concluded that propofol could sensitize doxorubicin-induced growth inhibition in osteosarcoma.

### 3.4. Propofol-Induced ER Stress in Tumour Models

**Figure 4:**
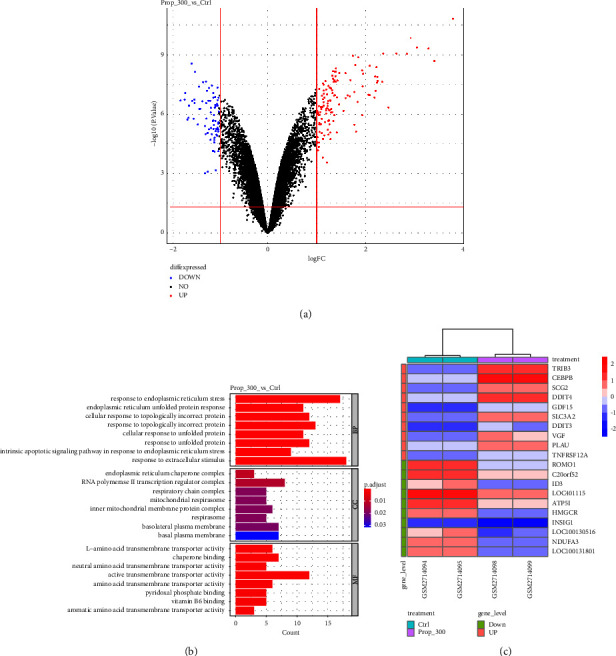
Bioinformatic analysis showed that propofol treatment led to endoplasmic reticulum stress. Transcriptional data of GSE101724 were downloaded from the GEO database and differentially expressed genes after propofol (300 *μ*M) treatment were functionally analysed. Differentially expressed genes were shown in the volcano plot (a) with up- and downregulated genes labelled as red or blue, respectively. GO functional enrichment of the differential expressed genes showed enrichment in the endoplasmic reticulum stress-related pathways (b). The top 10 up- or downregulated genes after propofol treatment were plotted as the heatmap (c).

In order to explore the molecular changes after propofol treatment, we analysed the transcriptional changes after propofol treatment in the dataset GSE101724. Raw data were downloaded from the GEO database, and the expression matrix was constructed. Differentially expressed genes were analysed between the propofol treatment group and the control group and plotted as a volcano plot (Figure 4(a)). Significantly differentially expressed genes were defined as |logFC| > 1 and *p* < 0.05. Altogether 195 genes met the criteria and were collected. Gene Ontology (GO) enrichment analysis on the differential expressed genes was performed, and the top 8 activities are plotted as shown in the bar graph (Figure 4(b)). The top 10 up- or downregulated genes based on logFC changes were plotted as a heatmap (Figure 4(c)). Interestingly, we found that most of the differential expressed genes were enriched in endoplasmic reticulum (ER) stress-related activities. ER stress is known to be involved in multiple tumour activities such as proliferation, invasion, and drug resistance. These data showed a correlation between propofol treatment with ER stress, which might explain our findings on the role of propofol in osteosarcoma in this study.

### 3.5. ER Stress Response Was Activated after Propofol Treatment in Osteosarcoma

**Figure 5:**
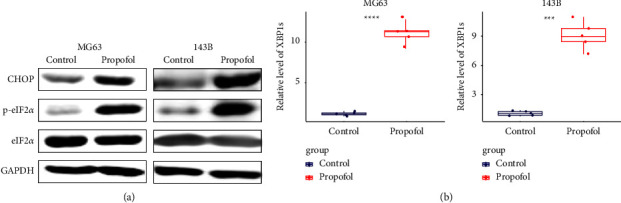
Propofol treatment-induced endoplasmic reticulum stress in osteosarcoma. The protein levels of CHOP and p-eIF2*α* were significantly increased after propofol treatment in MG63 and 143B (a). Upregulated mRNA expression of XBP1s was detected in MG63 and 143B after propofol treatment (b).

Next, we set out to test whether ER stress was involved in propofol treatment in osteosarcoma. We examined the expression of ER stress-related markers such as CHOP, p-eIF2*α*, and XBP1s. Western blot results showed that CHOP and p-eIF2*α* were upregulated after propofol treatment in both MG63 and 143B cells (Figure 5(a)). The presence of XBP1s was also detected in both cell lines after propofol treatment (Figure 5(b)). All these data suggested the concerted activation of all three ER stress sensors and their combinatorial response, validating that ER stress was induced by propofol treatment.

## 4. Discussion

In this study, we examined the antitumour role of propofol in osteosarcoma and explored the possible mechanism. We first tested the growth inhibition induced by propofol. CCK8 assay after propofol treatment showed significant growth inhibition in a dose-dependent manner in multiple osteosarcoma in vitro models. Tumour migration and invasion changes after propofol treatment were also assessed with the transwell assay. Results showed that propofol treatment induced decreased cell migration and invasion in both MG63 and 143B cell lines.

Next, we tried to test whether propofol could sensitize osteosarcoma to doxorubicin treatment. Proliferation assays of osteosarcoma cells cultured with different concentrations of doxorubicin with or without propofol were performed. Proliferation curves were plotted, and the IC50s under different conditions had been calculated to reflect the drug sensitivity. In accordance with similar experiments in other tumour types, propofol significantly reduced the IC50 and sensitized osteosarcoma to doxorubicin treatment.

We explored the possible mechanisms by evaluating the transcriptional changes after propofol treatment with bioinformatic tools. Differentially expressed genes were collected and functional enrichment highlighted ER stress response-related pathways. We were aware that the GSE101724 study was different from our study in tumour cell lines and propofol concentrations. GSE101724 study was used as pilot study to predict the possible roles of propofol on cells. The findings were validated in our study with osteosarcoma cell lines before jumping into any conclusion.

To validate the presence of ER stress and activation of unfolded protein response (UPR) after propofol treatment in osteosarcoma, all three pathways [20] were examined by testing the protein level of CHOP and p-eIF2*α* together with the presence of XBP1s mRNA. XBP1s is a truncate isoform produced by splicing of 26 nucleotides from the central part of the mRNA of XBP1. The production of XBP1s is mediated by the RNase domain of IRE1 after its multimerization under ER stress. The presence of the XBP1s mRNA supports the activation of IRE1 pathway under ER stress. Our data supported the activation of all three pathways and proved the presence of ER stress and UPR under propofol treatment. Previous studies have linked ER stress with propofol treatment. For example, propofol induces ER stress and autophagy by promoting calcium release and ROS production in C2C12 myoblast cell line [27]. Similar results were also observed in HeLa cells [28].

Limitations of this paper should be noticed. This study focused on the role of propofol on osteosarcoma and proposed the involvement of ER stress response; further studies are needed to fully illustrate the mechanisms on the detailed process of propofol-induced ER stress response. In addition, the antitumour role of propofol was based on the in vitro results of tumour cells treated with 2.5 *μ*g/ml of propofol for a duration of 48 and 72 hours. More studies should be conducted before we could find the appropriate usage of propofol in clinic as an antitumour agent.

## 5. Conclusions

In conclusion, we assessed the antitumour role of propofol on osteosarcoma and found that propofol could induce the ER stress response, inhibit osteosarcoma cell proliferation, migration, and invasion, and increase the sensitivity to doxorubicin treatment. Our findings provided a novel option in osteosarcoma therapy.

## Data Availability

The data used to support the findings of this study are included within the article.
